# Learning to Work Together—a BRIDGE to Engagement

**DOI:** 10.1007/s11606-021-07123-7

**Published:** 2021-09-13

**Authors:** Mary Ellen Houlihan

**Affiliations:** COPD Foundation, Miami, FL USA

The research community’s pivot and evolution, over the past decade, to a focus on patient-centered research topics, patient involvement in research development, and most recently the engagement of patients as full, contributing research team members, has been simultaneously unprecedented and the most natural progression of research history. As an individual with a chronic lung disease for over 20 years, often labeled as “patient,” I have watched and welcomed this evolution from both the sidelines and the trenches. However, while fully invested in the patient-centered, patient-engaged research initiatives, I can admit I was not always fully prepared for the research role I was asked to take on, needing, at times, more than the title of “patient” to feel fully comfortable, confident, and prepared to become engaged.

My view is not a singular one. The COPD Foundation is an organization focused on improving the lives of individuals with chronic obstructive pulmonary disease (COPD) for which I have served as patient investigator and overall advocate. This organization recognizes the gap that often exists for patients between their desire to participate and the confidence and comfort needed to actually raise their hand and join a team.

The Foundation, which has over 50,000 registered individuals in its patient community,^1^ understood that a comprehensive research-focused training for the COPD community could boost patients’/caregivers’ confidence, making them more likely to consider joining a research team while also improving their experience on the team. With that understanding, the Foundation focused on creating a research training program with a patient as co-lead of the team tasked with creating the training modules.

I am that patient co-lead, and the training program is the COPD Patient-Powered Research Network’s *BRIDGE Patient to Investigator Training*.^2^ The training consists of 11 online modules that provide fundamental research information, is focused on building confidence, and addresses the activity and mobility limitations within the COPD community, supporting those who might not be able to attend in-person trainings. I am excited to be a part of an initiative where I can use my own patient investigator experiences and lessons learned to create a training program that will encourage, educate, and ultimately motivate more patients to become involved in research, ensuring their voice is heard.

## LESSONS LEARNED: PRIOR PATIENT ENGAGEMENT EXPERIENCES

Prior to working on the BRIDGE Project, I was a member of the COPD Patient Powered Research Network (PPRN) Governing Board. The COPD PPRN is the COPD Foundation’s online registry of individuals with COPD and those with self-reported risk factors for COPD who have consented to be contacted about studies and complete surveys about their health and experience living with COPD. In my role as a governing board member, I was asked to serve as a patient investigator on the COPD INVEST Study,^3^ a collaborative effort of a large pharmaceutical company, a wearable device company, a health information technology and clinical research company, and the COPD Foundation to assess nighttime wearability of a biometric vest in people with COPD. My only qualifications for either of these roles are being a COPD patient and having a basic understanding of human subjects’ research.

## LEARNING A FOREIGN LANGUAGE

When I joined the PPRN Governing Board, I was given a great deal of information to bring me up-to-speed. Despite this thorough onboarding (a new term for me), I was faced with a steep learning curve—primarily because of terminology and acronyms. This happened again, to some degree, when I joined the COPD INVEST Study. Thank goodness for Google! Since joining the governing board, I have maintained a list of unfamiliar terms to use as a quick reference when participating in meetings. Every specialty has its own language, and any non-medical newcomer to research needs to learn that language if they are to fully understand and participate.

I took this knowledge and experience with me to my BRIDGE work. Developing a robust glossary and acronym list for our future BRIDGE trainees was something I was passionate about, and I made it one of my personal priorities as I worked with the BRIDGE team to create our lay-friendly, extensive research training program. The need for a robust glossary and list of acronyms for the BRIDGE training was further confirmed by many other patients and caregivers. These patients’/caregivers’ voices were heard through our BRIDGE Stakeholder Advisory Board (a group of COPD patients, caregivers, and COPD health care providers created to advise and provide feedback throughout the BRIDGE development process). Most of these patients/caregivers had lists of their own—collected through years of managing a chronic disease—we used these “road tested” lists to help us create our *BRIDGE Glossary* and *BRIDGE Acronym List*. The patients and caregivers on our Stakeholder Advisory Board have also tested and critiqued the training modules at critical times in the development process.

### Roles and Expectations

When I agreed to join the COPD INVEST Study, I did receive some information about what to expect; however, the actual team meetings were still overwhelming for me in the beginning. I was confused about the roles of all the people on the team, unsure of what my role was, and what was expected of me despite having personal confidence that came from a variety of careers and life experiences. Fortunately, the smaller COPD Foundation team working on the study met weekly to discuss action items and outstanding matters and I was able to ask questions to clarify roles and expectations. Not wanting other potential patient investigators to experience this, the BRIDGE team and I developed, for our Patient-to-Investigator training, a list of questions a patient might ask when considering joining a research team. The list includes questions about time commitment, logistics, compensation, and expectations, as well as general questions regarding terminology and the roles of all team members.

In addition, we have included, throughout the BRIDGE training modules, explanations and examples of how a patient investigator can contribute during different phases of research studies. Prior to creating the BRIDGE training, a literature review was done to determine what was already available. When I completed the existing trainings, I felt as though I had learned a great deal, but I was not envisioning myself on a research team nor motivated to become an investigator. With that in mind, we have ensured that the BRIDGE training includes specific, relatable examples of how patients can participate at each step of the research process. We have included explanations of why their contributions are important and what these contributions may look like during the many phases of a study from the planning to participant recruitment to evaluating and sharing results, in hopes of inspiring more patients to participate in the process.

### Providing Support

It had always seemed logical that including patients and caregivers on research teams would be beneficial, but I was surprised at how much the patient perspective could impact how a study progressed. Initially, I was intimidated by the size of the team and pace of the meetings for the INVEST Study. I was unsure if my observations were relevant and when and/or how to present them. Again, the weekly COPD Foundation team meetings were very helpful and gave me the opportunity to ask questions and voice my observations in a “safe” environment before speaking to the entire team. In the end, my suggestions were well-received by the entire team and led to significant adjustments regarding the sizing of the vests, the clarity of the patient-facing materials, and the logistics of when to conduct the trial. I believe the weekly COPD Foundation team meetings were the biggest factor in making my participation in the INVEST study a success. I used this experience to suggest and then help develop the concept of a Research Team Liaison as part of the BRIDGE training, a concept I think is particularly needed and will be beneficial for first-time patient investigators. The idea is that a research team member is designated as the patient investigator’s personal liaison throughout the study and should be available to answer the patient’s questions one-on-one, providing further clarification on topics, outside of the team’s regular meetings.

## IN CONCLUSION

In conclusion, I have enjoyed my own patient-investigator experience and find great satisfaction in knowing I contributed to and brought the patient perspective to important research. I have had the privilege of collaborating with the knowledgeable, caring COPD Foundation staff as well as other patients, caregivers, and stakeholders devoted to improving the lives of patients with COPD, and feel I am part of the solution and not just a casualty of this disease.

But beyond the satisfaction of this individual experience, I am most gratified to be able to work to improve the experience for future patient investigators through the careful development of the *BRIDGE Patient to Investigator* training. And most importantly, I hope this training will motivate more COPD patients and their caregivers to become active, participating members of research teams—ensuring their critical voice is heard.
